# Sexual dysfunction worsens both the general and specific quality of life of women with irritable bowel syndrome. A cross-sectional study

**DOI:** 10.1186/s12905-023-02272-9

**Published:** 2023-03-27

**Authors:** Santiago Camacho, Andrea Díaz, Paulina Pérez, Héctor Batalla, Yoshua Flores, Evelyn Altamirano, María de Fátima Higuera-de la Tijera, Daniel Murguía, Laura Gómez-Laguna

**Affiliations:** 1 Gastroenterology Service, Mexico General Hospital “Dr Eduardo Liceaga”, Dr. Balmis No 148, Col. Doctores. Alcaldía Cuauhtémoc, México City, 06720 Mexico; 2 Psychology Department, Iberoamerican University, México City, Mexico; 3 Higer Studies Faculty “Zaragoza”, National Autonomous University of Mexico, México City, Mexico; 4 Superior School of medicine, National Polytechnic Institute, México City, Mexico; 5 General Hospital of Zone #8, Mexican Social Security Institute, México City, Mexico; 6 Oncology Service, Mexico General Hospital “Dr Eduardo Liceaga”, México City, Mexico

**Keywords:** Irritable bowel syndrome, Sexual dysfunction, Quality of life, Comorbidity

## Abstract

**Background:**

Irritable bowel syndrome (IBS) and sexual dysfunction (SxD) lowers quality of life (QOL) separately, but the effect of their overlap in unselected populations has not been studied.

**Objective:**

To evaluate the QOL of IBS women with and without SxD and compare it with controls.

**Methods:**

In this cross-sectional assessment, we studied 51 IBS women (Rome IV criteria) and 54 controls. SxD was determined using the female sexual function index questionnaire. QOL was evaluated by the Short Form 36 (SF-36) and IBS-QOL questionnaires.

**Results:**

SxD prevalence was similar between IBS women (39.22%) and controls (38.89%). Compared with other groups, IBS patients with SxD showed lower scores in all domains as well as in the physical, mental summaries of the SF-36 and almost all domains (except for body image, food avoidance, and social reaction compared with IBS patients without SxD) and the total score of IBS-QOL.

**Conclusions:**

These findings show that SxD worsens both general and specific QOL of women with IBS. The consideration of SxD in patients with IBS will allow us to make a more effective diagnostic and therapeutic approach. Clinical trial registry in Mexico City General Hospital: DI/19/107/03/080. Clinical trials registration: NCT04716738.

**Supplementary Information:**

The online version contains supplementary material available at 10.1186/s12905-023-02272-9.

## Background

Although the use of different diagnostic criteria may prevent a complete understanding of its exact frequency, irritable bowel syndrome (IBS), a disorder of the brain-gut interaction, [1] affects between 3.8% (Rome IV criteria) and 9.2% (Rome III criteria) of the general population [2] and its frequency is almost two-fold (1.7 times) [3] increased in women compared with men. Only 67.7% of those affected seek information from their doctors, [4] but IBS still represents between 25% and 50% of gastroenterological consultations in the United States [5]. Regardless of the high prevalence of IBS reported in Mexico (up to 35.5%), [6]. its clinical presentation is similar to that observed worldwide.

IBS patients have more physical and psychological comorbidities, including sexual dysfunction (SxD) [7]. In 1987, Guthrie et al [8] reported that up to 83% of IBS patients might have SxD. A recent study in Poland demonstrated that up to 48% of IBS female patients referred to an outpatient clinic have SxD [9]. SxD is positively associated with the severity of gastrointestinal symptoms [10]. The problem is so significant that specific self-management programs for SxD in IBS patients have been developed [11]. Despite being one of the main comorbidities in IBS patients, studies on this topic are limited [12], and this deficiency must be corrected with primary research. Thus, there is a need to perform studies in populations where both conditions are present as a first approach to design appropriate therapeutic strategies aimed at improving medical care [13].

On the other hand, quality of life (QOL), which reflects subjective well-being, is reduced in patients with IBS [14] and SxD [15]. Recently, Sørensen et al [16] reported that SxD in IBS has been studied mostly as a secondary endpoint in QOL studies, so further studies are needed. Characterizing QOL in the unselected population suffering from both conditions would be meaningful. This study aimed to evaluate the QOL of IBS women and compare it with control populations both with and without SxD.

## Methods

### Subjects

This cross-sectional survey was conducted according to STROBE guidelines at the Gastroenterology Service, Mexico General Hospital “Eduardo Liceaga” and the Iberoamerican University following the Declaration of Helsinki and its subsequent declaration revisions. Informed consent was obtained from all subjects before the interview. This study, which investigates the prevalence of SxD in IBS patients as well as its influence on the quality of life in the Mexican female population, is part of a broader protocol that is ongoing, has been approved by the local ethics and research committees (DI/19/107/03/080) and is registered at clinicaltrials.gov (NCT04716738).

We recruited consecutive sexually active women in the open population by opportunity sampling from March to September 2019. For study inclusion, the participants must have met the following criteria: between 18 and 50 years, provided informed consent, and agreed to answer the questionnaires. We excluded women with noncontrolled systemic disease, pregnant women, breastfeeding women, menopausal women, women taking medications regularly in the previous 3 months, and women with an established diagnosis of other sexual and/or gastrointestinal diseases. Patients who did not accept contact information, did not answer the complete questionnaires, failed to understand the instructions, and/or were unreachable after the application of questionnaires were eliminated from the study.

To avoid selection bias, we applied the questionnaires in a single interview on consecutive women, and data were entered in a spreadsheet that was not analyzed immediately. We performed the first analysis 2 months after the start of the study that the psychology students presented at the Iberoamerican University. We performed a second analysis in July that was presented at the Mexican Congress of Gastroenterology. In November 2019, we performed the last interim analysis for the Digestive Disease Week. Finally, between July and December 2020, we performed the final analysis for this article.

### Irritable bowel syndrome diagnosis

We used the Rome IV criteria, which specify that abdominal pain is present for more than 6 months once a week in the last 12 and at least two of the following three characteristics are noted: (a) pain related to defecation associated with changes in the (b) frequency of defecation and/or in the (c) appearance of stool [17].

Using a pictorial representation of the Bristol Scale, we recorded the most frequent type of stool consistency subjectively perceived by the patient (Bristol Subjective Scale, BSS) and the percentage of time in which the patient objectively observed their different consistencies (Bristol Objective Scale, BOS). To determine the BSS score, we asked patients to select which of the drawings they perceived as the most frequent. After answering this question, we evaluated the BOS by asking the patient to provide the maximum number of recent bowel movements (they mentioned between 4 and 12) and characterize the feces according to the Bristol scale. Finally, we assigned a percentage to each type of evacuation. The following IBS subtypes were determined using the BOS as suggested by the Rome IV criteria: constipation (IBS-C), diarrhea (IBS-D), mixed (IBS-M), or undetermined (IBS-U) [17].

### Sexual dysfunction evaluation

We used the Female Sexual Function Index (FSFI), which has been validated in the Mexican population [18] and is considered the gold standard of female sexual functioning of women with gastrointestinal diseases [13]. With 19 items, the FSFI evaluates six domains: desire, excitement, lubrication, orgasm, satisfaction, and pain. A higher score indicates better function. Sexual dysfunction was noted when the total score (adding the score of each domain) was less than 26.55 [19].

### QOL measurements

Two instruments were used assessed the patient’s attitude toward her health status based on physical, psychological, and social aspects. We used the Short-Form 36 (SF-36) questionnaire for a general QOL evaluation based on eight domains: physical function, physical role, body pain, general health, vitality, social functioning, emotional role, and mental health. The questions were coded, summarized, and transformed to a scale from 0 (worst) to 100 (best) health statuses. Additionally, the questionnaire includes question about the change in the general health state. We also calculated physical (PCS) and mental (MCS) component summaries bases on an orthogonal approximation. This questionnaire has been validated in the Mexican [20] and IBS populations [14].

Specific QOL was assessed by the irritable bowel syndrome-quality of life (IBS-QOL) questionnaire [21]. This questionnaire evaluates eight domains (dysphoria, interference with activity, body image, health concern, food avoidance, social reaction, sexual concern, and social relationship), and a total score (the sum of all domains) is also provided. This questionnaire has been widely used in IBS [13]. Higher scores indicate better QOL.

### Sample size and power of the study

Because there are no previous articles that address the overlapping effects of IBS and SxD on QOL using the SF-36 and IBS-QOL as main outcomes in the same population, we decided to recruit consecutive women as the first approach. In a post hoc analysis (StatMate 2 for Windows, version 2.00), we found a beta power greater than 80% (with alpha < 0.05) on the size effect of the main variables (domains and total summaries of the SF-36 and IBS-QOL questionnaires), so we conclude that the sample size was sufficient to obtain statistical validity (Additional File 1 & 2).

### Statistical analysis

The main outcomes were the differences in domains, component summaries, and total scores of the SF-36 and IBS-QOL. Secondary outcomes included age, body mass index (BMI), marital status, pain related to menses, frequency of defecation per week, IBS subtype, and the Bristol Scale (subjective and objective). Statistical analysis was performed with IBM® SPSS® Statistics, version 26 (Registered for Iberoamerican University). When appropriate, data indicate the mean ± standard deviation, 95 % confidence intervals (CI), range, and/or percentages. Statistical differences were evaluated by Student’s t test for parametric data, Mann‒Whitney and chi-square tests for nonparametric data, and Fisher’s exact test with the Katz approximation for relative risk (RR). Alpha = 0.05 in these analyses. We analyzed the correlation between BSS and BOS through the Pearson test.

## Results

**Fig. 1:**
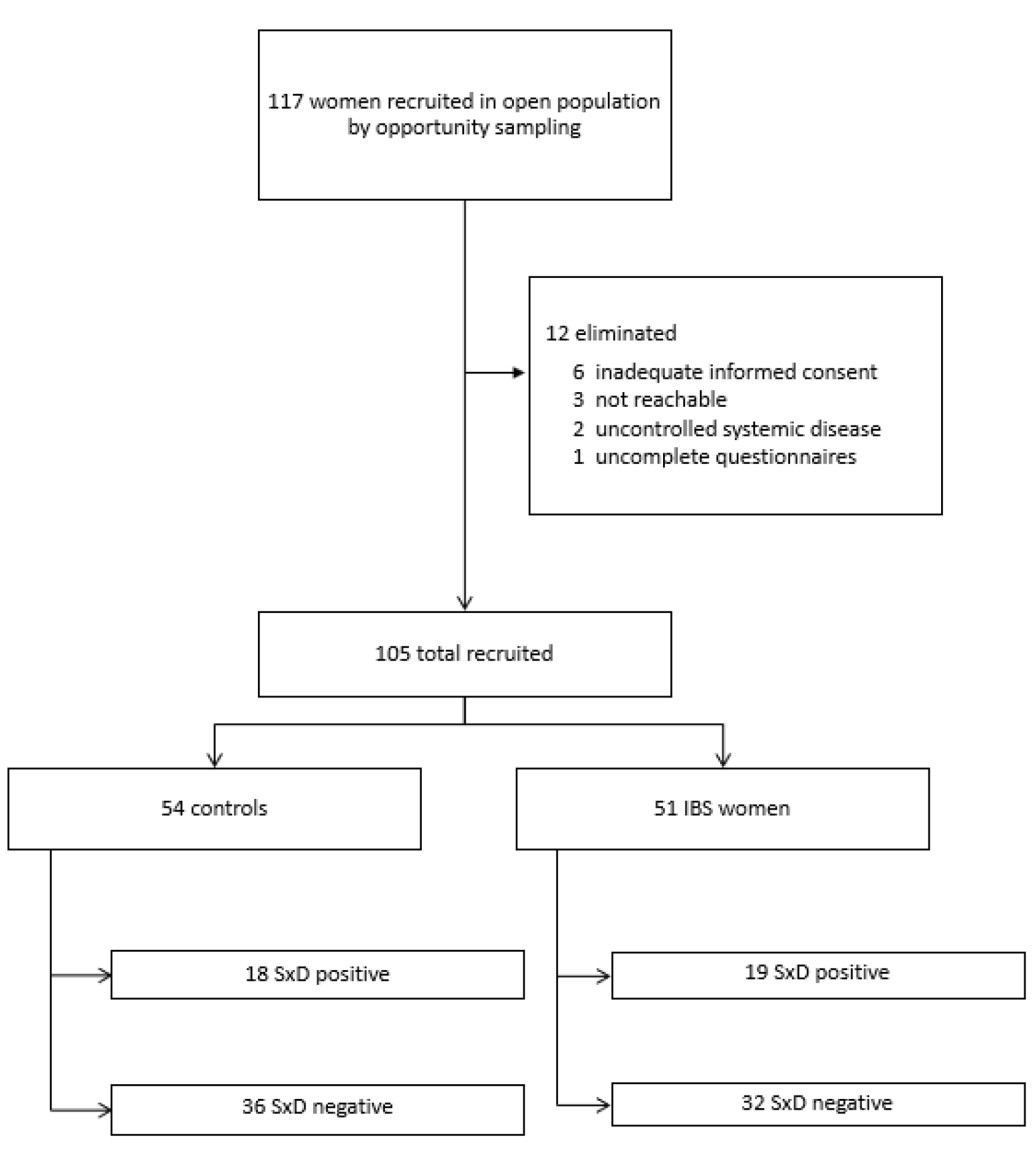
Similar prevalence of sexual dysfunction (SxD) between irritable bowel syndrome (IBS) women and controls in the non-selected population (37.25% vs. 33.33% respectively; p = 0.6, Fischer Exact test).

Of the 117 women recruited, 105 (89.7%) women were included in the study (see flowchart, Fig. 1). Age, BMI with cut points, pain related to menses, marital status of the total sample, and IBS and SxD patients are reported in Table 1. IBS women were older and more likely to be in a relationship. The weekly frequency of defecation was lower in IBS patients than in controls [9.33 ± 5.85 (7.73–10.94) vs. 13.15 ± 6.89 (11.31–14.99), respectively, p = 0.002] but similar in women with SxD versus those without SxD [11.24 ± 6.64 (9.21–13.28) vs. 11.15 ± 6.61 (9.58–12.72), respectively, p = 0.94].

**Table 1:** Age, BMI with cut points, pain related to menses, and marital status in total population, IBS, and SxD subgroups.

	Total (n = 105)	IBS (n = 51)	Controls (n = 54)	p	SxD (n = 37)	No SxD (n = 68)	p
Age (years)	26.65 ± 7.08 (25.29–28)	28.45 ± 7 (26.53–30.37)	24.94 ± 6.79 (23.13–26.76)	**0.0106**	27.76 ± 8.39 (25.05–30.46)	26.04 ± 6.25 (24.56–27.53)	0.2385
BMI (kg/m^2^)	22.14 ± 2.91 (21.59–22.7)	22.56 ± 2.45 (21.89–23.24)	21.75 ± 3.26 (20.87–22.62)	0.1506	21.59 ± 2.42 (20.81–22.37)	22.45 ± 3.13 (21.7–23.19)	0.1507
BMI cut points (%)							
Underweight (< 18)	6.67	1.90	4.76	0.6486	3.81	2.86	0.3078
Normal (18-24.9)	81.90	41.90	40.00		29.52	52.38	
Overweight I (25-29.9)	9.52	3.81	5.71		1.90	7.62	
Overweight II (30-39.9)	1.90	0.95	0.95		0.00	1.90	
Pain related to menses (%)	41.90	47.06	37.04	0.3277	37.84	44.12	0.6792
Marital status (%)							
Single	79.05	33.33	45.71	**0.0227**	24.76	54.29	0.1258
Married	14.29	8.57	5.71		5.71	8.57	
Divorced	1.90	1.90	0.00		1.90	0.00	
Free union	4.76	4.76	0.00		2.86	1.90	

Data express mean ± standard deviation (95%CI) or percentages as appropriate. IBS, irritable bowel syndrome, SxD, sexual dysfunction, BMI, body mass index. Fischer exact test or non-paired student *t*-test, two-sided. Significant statistical differences are bolded.

### Quality of life and sexual dysfunction in IBS women and controls

General and specific QOL as well as FSFI domains of IBS and SxD patients are shown in Table 2. No differences in the domains and total score of the FSFI were noted between IBS patients and controls.

**Table 2:** General (SF-36), specific (IBS-QOL) quality of life, and sexual function (FSFI) in total population, IBS and SxD subgroups.

	Total (n = 105)	IBS (n = 51)	Controls (n = 54)	p	SxD (n = 37)	No SxD (n = 68)	p
SF-36							
Physical Function	93.38 ± 13.02 (90.89–95.87)	90.49 ± 16.01 (86.1-94.88)	96.11 ± 8.67 (93.8-98.42)	**0.0263**	88.24 ± 17.61 (82.57–93.92)	96.18 ± 8.6 (94.13–98.22)	**0.0025**
Physical Role	83.1 ± 29.72 (77.41–88.78)	78.43 ± 32.02 (69.64–87.22)	87.5 ± 26.93 (80.32–94.68)	0.1186	77.7 ± 35.74 (66.19–89.22)	86.03 ± 25.69 (79.92–92.14)	0.1714
Body Pain	74.57 ± 21.83 (70.4-78.75)	66.72 ± 22.25 (60.61–72.82)	81.99 ± 18.79 (76.98-87)	**0.0002**	68.99 ± 24.05 (61.24–76.73)	77.61 ± 20.06 (72.84–82.38)	0.0527
General Health	64 ± 18.7 (60.42–67.58)	58.63 ± 18.28 (53.61–63.64)	69.07 ± 17.81 (64.32–73.82)	**0.0037**	59.19 ± 17.18 (53.65–64.72)	66.62 ± 19.09 (62.08–71.16)	0.0514
Vitality	52.48 ± 16.44 (49.33–55.62)	50 ± 18.3 (44.98–55.02)	54.81 ± 14.24 (51.02–58.61)	0.1343	49.19 ± 15.75 (44.12–54.26)	54.26 ± 16.65 (50.31–58.22)	0.1314
Social Functioning	74.88 ± 22.3 (70.62–79.15)	68.14 ± 23.76 (61.62–74.66)	81.25 ± 18.93 (76.2–86.3)	**0.0022**	67.23 ± 22.51 (59.98–74.48)	79.04 ± 21.21 (74-84.09)	**0.0088**
Emotional Role	63.81 ± 41.63 (55.85–71.77)	53.59 ± 42.73 (41.87–65.32)	73.46 ± 38.52 (63.18–83.73)	**0.0138**	53.15 ± 41.91 (39.65–66.66)	69.61 ± 40.62 (59.95–79.26)	0.0526
Mental Health	67.7 ± 15 (64.83–70.57)	64.78 ± 16.57 (60.24–69.33)	70.44 ± 12.92 (67-73.89)	0.0529	63.03 ± 14.62 (58.32–67.74)	70.24 ± 14.7 (66.74–73.73)	**0.0180**
Physical Component Score (PCS)	59.45 ± 10.63 (57.42–61.49)	56.21 ± 11.28 (53.12–59.31)	62.51 ± 9.06 (60.1-64.93)	**0.0020**	56 ± 11.54 (52.28–59.72)	61.33 ± 9.67 (59.03–63.63)	**0.0133**
Mental Component Score (MCS)	45.16 ± 5.93 (44.02–46.29)	44.11 ± 6.78 (42.25–45.97)	46.14 ± 4.86 (44.85–47.44)	0.0791	43.89 ± 5.83 (42.01–45.77)	45.85 ± 5.91 (44.44–47.25)	0.1061
Health change (1 year, %)							
Better/much better	32.38	12.38	20.00	**0.0092**	9.52	22.86	**0.0097**
Same	54.29	24.76	29.52		16.19	38.10	
Worst/much worst	13.33	11.43	1.90		9.52	3.81	
IBS-QOL							
Dysphoria	87.62 ± 19.75 (83.84–91.4)	79.72 ± 23.99 (73.13–86.3)	95.08 ± 10.29 (92.34–97.83)	**0.0000**	79.9 ± 24.88 (71.88–87.91)	91.82 ± 14.91 (88.28–95.36)	**0.0027**
Interference with the activity	86.19 ± 19.77 (82.41–89.97)	77.87 ± 22.63 (71.66–84.08)	94.05 ± 12.41 (90.74–97.36)	**0.0000**	78.28 ± 23.65 (70.66–85.9)	90.49 ± 15.9 (86.72–94.27)	**0.0021**
Body image	80.24 ± 21.32 (76.16–84.32)	69.73 ± 22.34 (63.6-75.86)	90.16 ± 14.65 (86.25–94.07)	**0.0000**	75 ± 25.04 (66.93–83.07)	83.09 ± 18.58 (78.67–87.5)	0.0630
Health concern	79.13 ± 25.19 (74.31–83.95)	66.34 ± 27.13 (58.89–73.79)	91.2 ± 15.65 (87.03–95.38)	**0.0000**	72.07 ± 30.25 (62.32–81.82)	82.97 ± 21.23 (77.92–88.01)	**0.0336**
Food avoidance	72.86 ± 27.71 (67.56–78.16)	58.01 ± 29.39 (49.94–66.07)	86.88 ± 16.71 (82.42–91.34)	**0.0000**	65.54 ± 34.37 (54.47–76.61)	76.84 ± 22.62 (71.46–82.21)	**0.0454**
Social reaction	85.65 ± 21.41 (81.56–89.75)	77.33 ± 24.87 (70.5-84.15)	93.52 ± 13.63 (89.88–97.15)	**0.0001**	78.89 ± 25.36 (70.71–87.06)	89.34 ± 18.07 (85.04–93.63)	**0.0161**
Sexual concern	91.07 ± 17.91 (87.65–94.5)	85.05 ± 22.5 (78.87–91.22)	96.76 ± 9.15 (94.32–99.2)	**0.0006**	85.14 ± 21.62 (78.17–92.1)	94.3 ± 14.72 (90.8–97.8)	**0.0115**
Social relationship	86.9 ± 21.27 (82.84–90.97)	79.25 ± 24.8 (72.44–86.05)	94.14 ± 14.08 (90.38–97.89)	**0.0002**	80.18 ± 25.64 (71.92–88.44)	90.56 ± 17.63 (86.37–94.75)	**0.0161**
Overall Score	84.31 ± 18.89 (80.7-87.93)	75.06 ± 21.29 (69.21–80.9)	93.06 ± 10.59 (90.23–95.88)	**0.0000**	77.25 ± 23.68 (69.62–84.88)	88.16 ± 14.49 (84.72–91.6)	**0.0042**
FSFI							
Desire	3.82 ± 1.16 (3.59–4.04)	3.82 ± 1.05 (3.53–4.11)	3.81 ± 1.27 (3.47–4.15)	0.9567	2.89 ± 1.23 (2.49–3.28)	4.32 ± 0.74 (4.15–4.5)	**0.0000**
﻿ Excitement	4.19 ± 1.75 (3.86–4.53)	4.34 ± 1.47 (3.93–4.74)	4.06 ± 1.98 (3.53–4.58)	0.4146	2.44 ± 1.81 (1.86–3.03)	5.14 ± 0.59 (5-5.28)	**0.0000**
﻿ Lubrication	4.26 ± 1.84 (3.91–4.61)	4.46 ± 1.58 (4.03–4.9)	4.07 ± 2.04 (3.53–4.62)	0.2756	2.51 ± 2 (1.87–3.16)	5.21 ± 0.69 (5.05–5.38)	**0.0000**
﻿ Orgasm	4.09 ± 1.96 (3.72–4.47)	4.29 ± 1.74 (3.81–4.77)	3.9 ± 2.15 (3.33–4.48)	0.3150	2.14 ± 1.91 (1.53–2.76)	5.15 ± 0.87 (4.95–5.36)	**0.0000**
﻿ Satisfaction	4.7 ± 1.27 (4.46–4.95)	4.72 ± 1.25 (4.38–5.06)	4.69 ± 1.3 (4.34–5.04)	0.8958	3.51 ± 1.27 (3.1–3.92)	5.35 ± 0.65 (5.2–5.51)	**0.0000**
﻿ Pain	4.05 ± 2.01 (3.66–4.43)	4.38 ± 1.74 (3.9–4.85)	3.74 ± 2.21 (3.15–4.33)	0.1057	2.11 ± 2.12 (1.42–2.79)	5.11 ± 0.8 (4.91–5.3)	**0.0000**
﻿ Total Score	25.12 ± 8.69 (23.46–26.78)	26.01 ± 7.45 (23.97–28.06)	24.27 ± 9.71 (21.68–26.86)	0.3074	15.6 ± 7.95 (13.04–18.16)	30.29 ± 2.45 (29.71–30.88)	**0.0000**

Data express mean ± standard deviation (95%CI) or percentages as appropriate. IBS = Irritable Bowel Syndrome, SxD = Sexual dysfunction. Fischer Exact test or Non-Paired Student *t*-Test, two-sided. Significant statistical differences are bolded.

#### General QOL (SF-36)

The IBS women in our study showed lower scores in the domains of body pain, general health, and emotional role, whereas SxD women exhibited a difference in mental health. Patients with both diseases have lower scores for physical function, social functioning, PCS, and deterioration in health compared with the previous year.

#### Specific QOL (IBS-QOL)

IBS women scored lower on the eight domains and the total score, whereas SxD patients scored lower on seven domains (except body image) and the total score.

#### Overlap of IBS and SxD

IBS patients without SxD exhibited a higher BMI than SxD women. Patients with both diseases were older than controls, reported lower PCS and MCS scores, and showed health deterioration (Table 3). The IBS-QOL total score was lower in IBS women than controls. In addition, IBS women with SxD showed the lowest score even compared with IBS patients without SxD.

**Table 3:** Age, body mass index (BMI), physical and mental components scores and health change of the SF-36 and total score for IBS-QOL questionnaire between irritable bowel syndrome (IBS) women, controls (Ctl) with and without Sexual Dysfunction (SxD) subgroups.

	IBS with SxD (n = 19)	IBS without SxD (n = 32)	Ctl with SxD (n = 18)	Ctl without SxD (n = 36)
Age (years)	30.42 ± 9.09 (26.33–34.51)	27.28 ± 5.22 (25.47–29.09)	24.94 ± 6.72 (21.84–28.05)*	24.94 ± 6.93 (22.68–27.21)*
BMI (Kg/m^2^)	21.68 ± 2.28 (20.66–22.7)#	23.09 ± 2.43 (22.25–23.93)	21.49 ± 2.62 (20.28–22.7)#	21.87 ± 3.57 (20.71–23.04)
SF-36				
Physical Component Score	50.05 ± 10.2 (45.46–54.64)	59.87 ± 10.37 (56.28–63.47)*	62.28 ± 9.51 (57.89–66.67)*	62.63 ± 8.96 (59.71–65.56)*
Mental Component Score	41.51 ± 5.74 (38.93–44.09)	45.65 ± 6.96 (43.24–48.07)*	46.4 ± 4.92 (44.12–48.67)*	46.02 ± 4.89 (44.42–47.61)*
Health change (1 year, %)				
Better/much better	15.79	31.25	38.89	38.89
﻿Same	36.84	59.38	55.56	58.33
﻿Worst/much worst	47.37	9.38*	5.56*	2.78*
IBS-QOL				
Total Score	65.05 ± 24.23 (54.16–75.95)	81 ± 17.11 (75.07–86.93)*	90.11 ± 14.98 (83.19–97.03)*#	94.53 ± 7.36 (92.12–96.93)*#

Data express Mean ± Standard Deviation (95%CI). Fischer Exact test or Non-Paired Student *t*-Test, two-sided. * p < 0.05 vs. IBS with SxD. # p < 0.05 vs. IBS without SxD.

**Fig. 2:**
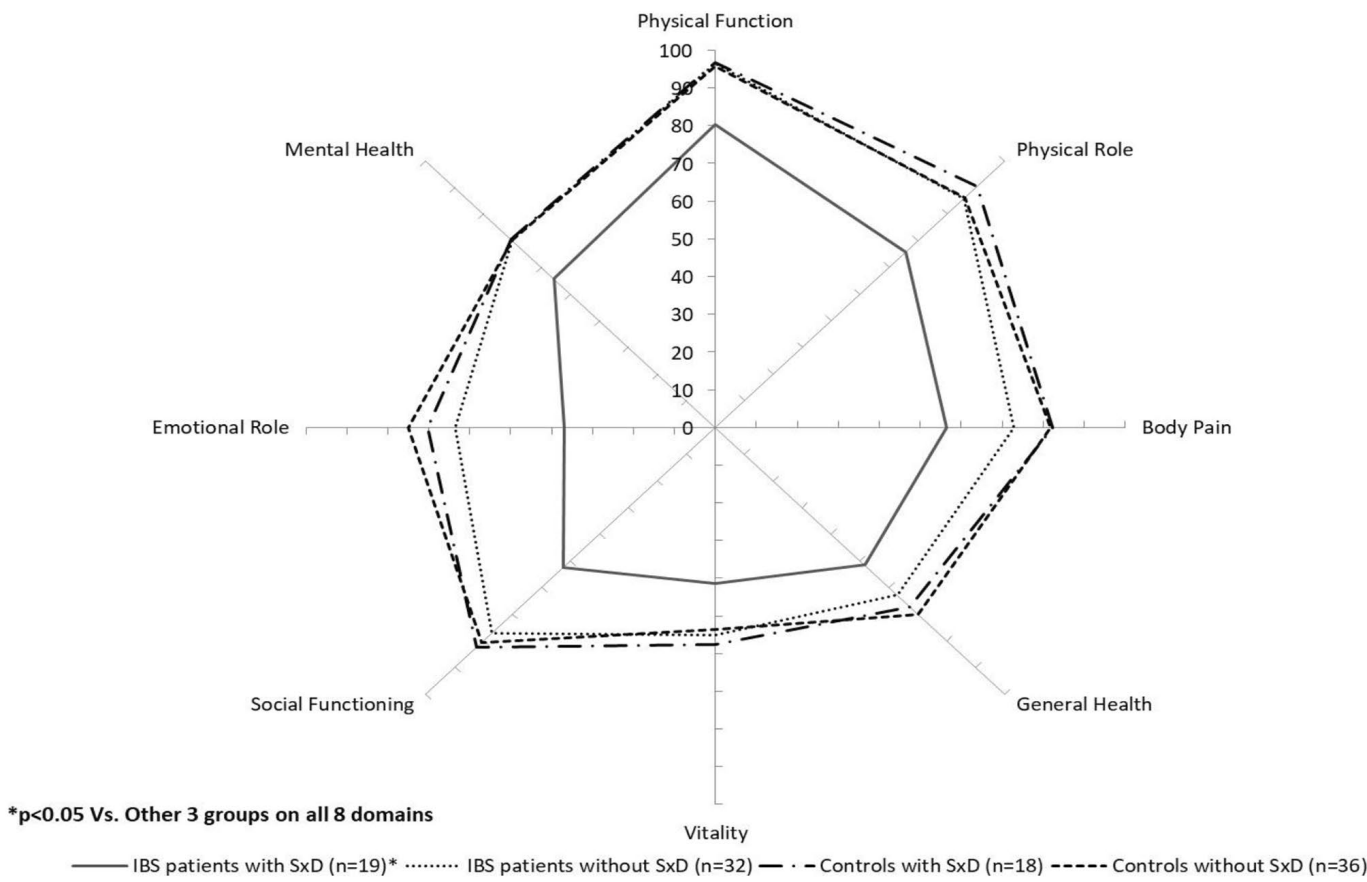
General quality of life (SF-36) in women with irritable bowel syndrome (IBS) and controls with and without sexual dysfunction (SxD).

**Fig. 3:**
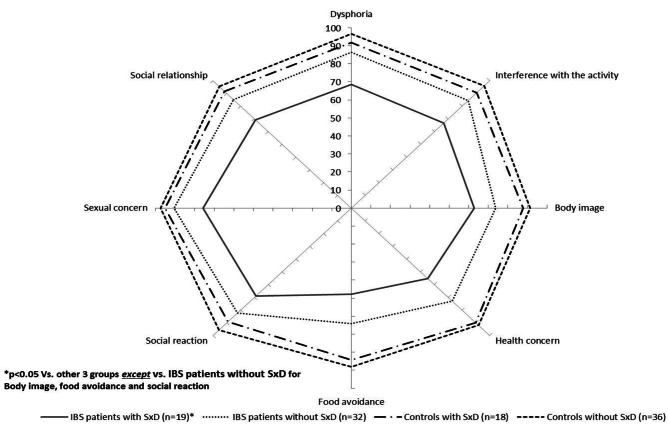
Specific quality of life (IBS-QOL) in women with irritable bowel syndrome (IBS) and controls with and without sexual dysfunction (SxD).

Patients with IBS and SxD scored lower on the eight domains of the SF-36 (Fig. 2) and five domains (except for body image, food avoidance, and social reaction that showed no differences compared with IBS women without SxD) of the IBS-QOL (Fig. 3).

### IBS subtype analysis

Twenty-four (47.06%) patients were classified as IBS-U type, 20 (39.22%) were IBS-C, and 7 (13.73%) were IBS-D. No IBS-M patients were identified. No differences (p > 0.05) in SxD prevalence were found among IBS-U (29.17%), IBS-C (45%), and IBS-D (42.86%). IBS-C patients obtained lower scores on the physical function, general health, vitality, social functioning, and mental health domains than IBS-U patients (Additional File 3).

PCS was higher in IBS-U [61.7 ± 8.47 (58.32–65.09)] than in IBS-C [50.62 ± 11.94 (45.38–55.85), p = 0.0008] but not in IBS-D [53.38 ± 9.94 (46.02–60.74), p = 0.5]. No differences in MCS (p > 0.05) were noted among IBS-U [44.82 ± 6.77 (42.12–47.53)], IBS-D [45.29 ± 5.26 (41.4–49.19)], and IBS-C [42.84 ± 7.35 (39.62–46.06)]. Worse/much worse change in health perception within one year did not differ (p > 0.05) among the IBS-U (12.5%), IBS-D (28.57%) or IBS-C (35%) groups.

Additional File 4 shows the lower specific quality of life of the eight domains in IBS-C women compared with IBS-U women. The total score was lower in IBS-C [64.89 ± 25.26 (53.82–75.96)] than in IBS-U [85.72 ± 10.41 (81.56–89.89), p = 0.0006] but not in IBS-D [67.54 ± 21.83 (51.37–83.71), p = 0.8].

Concordance, which is defined as the percentage of times in which the BSS and BOS were the same between subjects in each group, was good/excellent (higher than 80%, p > 0.3, chi square test) in the total population (84%), patients with IBS (84%) and controls (83%). Similarly, the total population (r = 0.83), IBS patients (r = 0.9) and controls (r = 0.72) showed a direct correlation (p < 0.01) between BSS and BOS.

### Predictive factors

Singleness decreased the RR of IBS by 0.772 (p = 0.01) but not for SxD (RR = 0.626, p = 0.1). BMI did not affect IBS (RR = 1.3, p = 0.3) or SxD (RR = 1.1, p = 0.7) women. Pain related to menses did not differ between the IBS versus control (47.06% vs. 37.04%, respectively; RR = 0.8, p = 0.3) or SxD positive and negative (37.84% vs. 44.12%, respectively; RR = 1.1, p = 0.6) groups. BSS types 3, 4 and 5 were protective factors associated with IBS compared with controls (62.75% vs. 87.04%, respectively; RR = 0.5543, p = 0.006).

## Discussion

Our study suggests that SxD worsens both the general and the specific QOL of female IBS patients. We found that approximately three out of ten women had SxD (IBS and control), which is lower than that previously reported (48%) [9]. We believe that our study more accurately reflects the prevalence of SxD because the previous study recruited patients from an outpatient clinic, whereas we recruited patients from an open population.

IBS women were older than controls. These findings are not consistent with the literature data that show a small protective factor in individuals older than 50 years [22]. Apart from the fact that our population was younger than 50 years, a recent review shows that this difference is maintained in Western countries but not in Taiwan, suggesting that an alternate component (such as comorbidity) is involved [23]. Our analysis is congruent with this finding because this difference disappears when comparing patients with IBS alone and controls, suggesting that SxD occurs more frequently in older IBS patients. Although IBS patients without SxD exhibited a higher BMI compared with IBS patients with SxD, BMI was in the normal range in all groups, which is consistent with the literature [24].

Pain related to menses did not differ among the groups. Premenopausal women with IBS report worsening of symptoms during menstruation compared with their follicular phase [25]. This finding is similar to that noted in healthy controls, who report abdominal pain more frequently during menses [26]. These manifestations are believed to be caused by sexual hormonal changes. We did not compare sexual cycle phases; however, we can infer that the decrease in QOL is not associated with pain related to menses.

IBS is more common in women who have had or are currently in relationships. This is finding is consistent with a study that found that married individuals had an increased odds ratio of IBS compared with nonmarried individuals (OR = 1.19) [27]. To our knowledge, this is the first report to describe being single as a protective factor (22.7%) for developing IBS.

Our IBS patients showed a lower weekly frequency of defecation, which is consistent with alterations in bowel movement as a diagnostic feature of IBS [17]. Interestingly, this difference disappears in SxD women. This finding potentially indicates that the frequency of defecation does not influence SxD, a finding not reported hitherto. Consistently, no differences in FSFI questionnaire scores were noted among IBS patients, which suggests that SxD is not influenced by IBS in a nonselected population; however, lower scores have been reported for IBS outpatients [9]. This difference may be explained by the fact that, for example, IBS-C outpatients are predisposed to report comorbidities, such as inflammatory bowel disease, more frequently to an obstetrician/gynecologist than other conditions [4].

A direct correlation was noted between subjective perception and objective recollection of fecal consistency, which validates its clinical use. This outcome is consistent with previously reported findings [28]. We identified a protective factor of BSS 3, 4 and 5 (50%) for IBS, a finding not reported previously.

Comparing the general QOL of IBS and SxD women separately, these patient groups share a deterioration in physical function and PCS, suggesting a common pathophysiological characteristic that would explain the poor subjective well-being perception. These findings agree with a recent study of patients with major depressive disorder treated with antidepressant drugs in which symptom responders showed an increase in functional connectivity in the dorsal anterior cingulate cortex (dACC) relative to nonresponders with improvement in physical QOL [29]. It has been demonstrated that SxD decreases brain activity of the dACC, [30] and IBS patients also show a decreased amplitude of low-frequency fluctuation in the dACC [31]. We believe this is an interesting findings that should be assessed in future research [32]. Lower IBS-QOL scores were noted in IBS patients, and similar scores were noted in SxD women (except for body image). This finding is consistent with those previously reported [16].

QOL is particularly important for IBS not only as a diagnostic or pathophysiological feature but also as a prognostic effectiveness factor. The latest clinical guidelines consider QOL and comorbidity as indicators for healthcare-seeking behavior and consequently as a diagnostic-therapeutic goal [33]. Our study adds a supporting feature to this strategy. Because SxD significantly decreases the general and specific quality of life, we should change the objectives to treat SxD simultaneously as the main comorbidity of women with IBS to adopt an approach based on patient-centered medicine as a recent review suggests [34].

IBS subtype analysis shows a similar prevalence of SxD, but IBS-C patients scored lower on five domains compared with IBS-U patients. Given the small sample size, we are unable to make a definitive conclusion, but this approach will be interesting to investigate in future work.

This research is the first step to elucidate the subjective perception of well-being as a diagnostic, therapeutic and prognostic objective. The clinical implications of these results are related to health-seeking behavior [35] and are thus important for providing a more precise determination of disease prevalence and incidence that could alter management strategies in women with IBS and SxD simultaneously.

Our study has some limitations to consider. We did not perform an exhaustive evaluation of clinical IBS or SxD. Although the diagnosis of IBS is clinical and the Rome IV criteria are sufficient to diagnose it, the possibility that abdominal pain may be secondary to another disease is latent. If we do not make a general laboratory evaluation, we will not be certain of the presence of IBS. Similarly, SxD requires a thorough psychiatric evaluation to determine which of the possible subtypes is present. Our approach assessed individuals from an open population, so we only trusted what each person reported and used self-administered questionnaires. Another limitation is that the power of prediction in cross-sectional studies is weak; thus, the RR results only serve as an approximation that should be validated in subsequent prospective studies.

## Conclusion

Sexual dysfunction could worsen both the general and the specific quality of life of women with irritable bowel syndrome. Further investigations of clinical and pathophysiological mechanisms are needed to elucidate the relationship between both diseases and determine whether these diseases share a common mechanism that explains this low quality of life. Considering sexual dysfunction in women with irritable bowel syndrome will allow us to provide a more effective diagnostic and therapeutic patient-centered approach.

## Electronic supplementary material

Below is the link to the electronic supplementary material.

Additional File 1: Comparison of the domains of the SF-36 by subgroups with p values

Additional File 2: Comparison of the domains of the IBS-QOL by subgroups with p values

Additional File 3: General Quality of life (SF-36) of women with irritable bowel syndrome (IBS) compared by subtypes

Additional File 4: Specific Quality of Life (IBS-QOL) in women with irritable bowel syndrome (IBS) compared by subtypes

## Data Availability

The datasets used and/or analyzed during the current study available from the corresponding author on reasonable request.

## List of abbreviations

IBS: Irritable Bowel Syndrome
SxD: Sexual Dysfunction
QOL: Quality of Life
SF-36: Short Form 36 questionnaire
IBS-QOL: Irritable Bowel Syndrome-Quality of Life questionnaire
BSS: Bristol Subjective Scale
BOS: Bristol Objective Scale
dACC: Dorsal Anterior Cingulate Cortex.
