# Supplementary material for: Sexual dysfunction worsens both the general and specific quality of life of women with irritable bowel syndrome. A cross-sectional study
Source: BMC Womens Health. 2023 Mar 27;23:134. doi: 10.1186/s12905-023-02272-9 (PMC10045848; doi:10.1186/s12905-023-02272-9)
Supplement: Supplementary file 1 — Additional File 1: Comparison of the domains of the SF-36 by subgroups with p values [file 12905_2023_2272_MOESM1_ESM.docx]

**Statistical considerations**

**Supplementary table A.** Comparison of the domains of the SF-36 by subgroups with p values. The beta power calculation is >80% for all domains.

|  | Physical Function | Role-Physical | Body Pain | General Health | Vitality | Social Functioning | Role Emotional | Mental Health | PCS | MCS |
| --- | --- | --- | --- | --- | --- | --- | --- | --- | --- | --- |
| IBS patients |  |  |  |  |  |  |  |  |  |  |
| With SxD (n=19) | 80.26±21.31  (70.68-89.84) | 65.79±38.38  (48.53-83.05) | 56.45±25.61  (44.93-67.96) | 51.58±12.25  (46.07-57.09) | 41.32±14.61  (34.75-47.89) | 52.63±18.9  (44.13-61.13) | 36.84±38.32  (19.61-54.07) | 55.79±14.7  (49.18-62.4) | 50.05±10.2  (45.46-54.64) | 41.51±5.74  (38.93-44.09) |
| Without SxD (n=32) | 96.56±6.89  (94.18-98.95) | 85.94±25.35  (77.15-94.72) | 72.81±17.73  (66.67-78.96) | 62.81±20.08  (55.86-69.77) | 55.16±18.51  (48.74-61.57) | 77.34±21.64  (69.85-84.84) | 63.54±42.64  (48.77-78.31) | 70.13±15.44  (64.77-75.48) | 59.87±10.37  (56.28-63.47) | 45.65±6.96  (43.24-48.07) |
| Controls |  |  |  |  |  |  |  |  |  |  |
| With SxD (n=18) | 96.67±5.42  (94.16-99.17) | 90.28±28.62  (77.06-103.5) | 82.22±13  (76.22-88.23) | 67.22±18.25  (58.79-75.65) | 57.5±12.51  (51.72-63.28) | 82.64±14.31  (76.03-89.25) | 70.37±39.42  (52.16-88.58) | 70.67±10.17  (65.97-75.37) | 62.28±9.51  (57.89-66.67) | 46.4±4.92  (44.12-48.67) |
| Without SxD (n=36) | 95.83±9.96  (92.58-99.09) | 86.11±26.35  (77.5-94.72) | 81.88±21.27  (74.93-88.82) | 70±17.77  (64.2-75.8) | 53.47±15.02  (48.57-58.38) | 80.56±21.01  (73.69-87.42) | 75±38.52  (62.42-87.58) | 70.33±14.23  (65.69-74.98) | 62.63±8.96  (59.71-65.56) | 46.02±4.89  (44.42-47.61) |
|  | **p Values (Student t Test)** | | | | | | | |  |  |
| IBS & SxD patients vs: |  |  |  |  |  | |  |  |  |  |
| IBS (+) SxD (-) | 0.0002 | 0.0283 | 0.0096 | 0.0324 | 0.0077 | 0.0001 | 0.0295 | 0.0020 | 0.0019 | 0.0333 |
| Controls SxD (+) | 0.0032 | 0.0352 | 0.0005 | 0.0040 | 0.0010 | 0.0000 | 0.0128 | 0.0011 | 0.0006 | 0.0088 |
| Controls SxD (-) | 0.0005 | 0.0246 | 0.0003 | 0.0002 | 0.0057 | 0.0000 | 0.0010 | 0.0008 | 0.0000 | 0.0035 |

Data express mean±standard deviation (95%CI). IBS, irritable bowel syndrome, SxD, sexual dysfunction, PCS, physical component summary and MCS, mental component summary.
