# Supplementary material for: Sexual dysfunction worsens both the general and specific quality of life of women with irritable bowel syndrome. A cross-sectional study
Source: BMC Womens Health. 2023 Mar 27;23:134. doi: 10.1186/s12905-023-02272-9 (PMC10045848; doi:10.1186/s12905-023-02272-9)
Supplement: Supplementary file 2 — Additional File 2: Comparison of the domains of the IBS-QOL by subgroups with p values [file 12905_2023_2272_MOESM2_ESM.docx]

**Statistical considerations**

**Supplementary table B.** Comparison of the domains of the IBS-QOL by subgroups with p values. The beta power calculation is >80% for all domains.

|  | Dysphoria | Interference with the activity | Body image | Health concern | Food avoidance | Social reaction | Sexual concern | Social relationship | Overall score |
| --- | --- | --- | --- | --- | --- | --- | --- | --- | --- |
| IBS patients |  |  |  |  |  |  |  |  |  |
| With SxD (n=19) | 68.59±26.81  (56.53-80.64) | 66.73±23.09  (56.35-77.11) | 62.83±26.47  (50.93-74.73) | 55.26±30.46  (41.57-68.96) | 47.81±36.09  (31.58-64.04) | 69.08±26.72  (57.07-81.09) | 75.66±24.46  (64.66-86.66) | 69.3±25.31  (57.92-80.68) | 65.05±24.23  (54.16-75.95) |
| Without SxD (n=32) | 86.33±19.74  (79.49-93.17) | 84.49±19.89 (77.6-91.38) | 73.83±18.75  (67.33-80.32) | 72.92±22.99  (64.95-80.88) | 64.06±23.13  (56.05-72.08) | 82.23±22.74  (74.35-90.1) | 90.63±19.57  (83.84-97.41) | 85.16±22.87  (77.23-93.08) | 81±17.11  (75.07-86.93) |
| Controls |  |  |  |  |  |  |  |  |  |
| With SxD (n=18) | 91.84±15.98  (84.46-99.22) | 90.48±17.71  (82.29-98.66) | 87.85±15.68  (80.61-95.09) | 89.81±17.52  (81.72-97.91) | 84.26±19.99  (75.03-93.49) | 89.24±19.62  (80.17-98.3) | 95.14±12.23  (89.49-100.79) | 91.67±21  (81.96-101.37) | 90.11±14.98  (83.19-97.03) |
| Without SxD (n=36) | 96.7±5.33  (94.96-98.44) | 95.83±8.43  (93.08-98.59) | 91.32±14.2  (86.68-95.96) | 91.9±14.84  (87.05-96.75) | 88.19±14.96  (83.31-93.08) | 95.66±8.94  (92.74-98.58) | 97.57±7.21  (95.21-99.92) | 95.37±9.01  (92.43-98.31) | 94.53±7.36  (92.12-96.93) |
|  | **p Values (Student t Test)** | | | | | | | |  |
| IBS & SxD patients vs: |  |  |  |  |  | |  |  |  |
| IBS (+) SxD (-) | 0.0092 | 0.0055 | 0.0892 | 0.0231 | 0.0553 | 0.0675 | 0.0201 | 0.0257 | 0.0083 |
| Controls SxD (+) | 0.0031 | 0.0013 | 0.0014 | 0.0002 | 0.0006 | 0.0135 | 0.0045 | 0.0061 | 0.0006 |
| Controls SxD (-) | 0.0000 | 0.0000 | 0.0000 | 0.0000 | 0.0000 | 0.0000 | 0.0000 | 0.0000 | 0.0000 |

Data express mean±standard deviation (95%CI). IBS, irritable bowel syndrome, SxD, sexual dysfunction.
