# Supplementary material for: Sexual dysfunction worsens both the general and specific quality of life of women with irritable bowel syndrome. A cross-sectional study
Source: BMC Womens Health. 2023 Mar 27;23:134. doi: 10.1186/s12905-023-02272-9 (PMC10045848; doi:10.1186/s12905-023-02272-9)
Supplement: Supplementary file 3 — Additional File 3: General Quality of life (SF-36) of women with irritable bowel syndrome (IBS) compared by subtypes [file 12905_2023_2272_MOESM3_ESM.docx]

Supplementary figure C
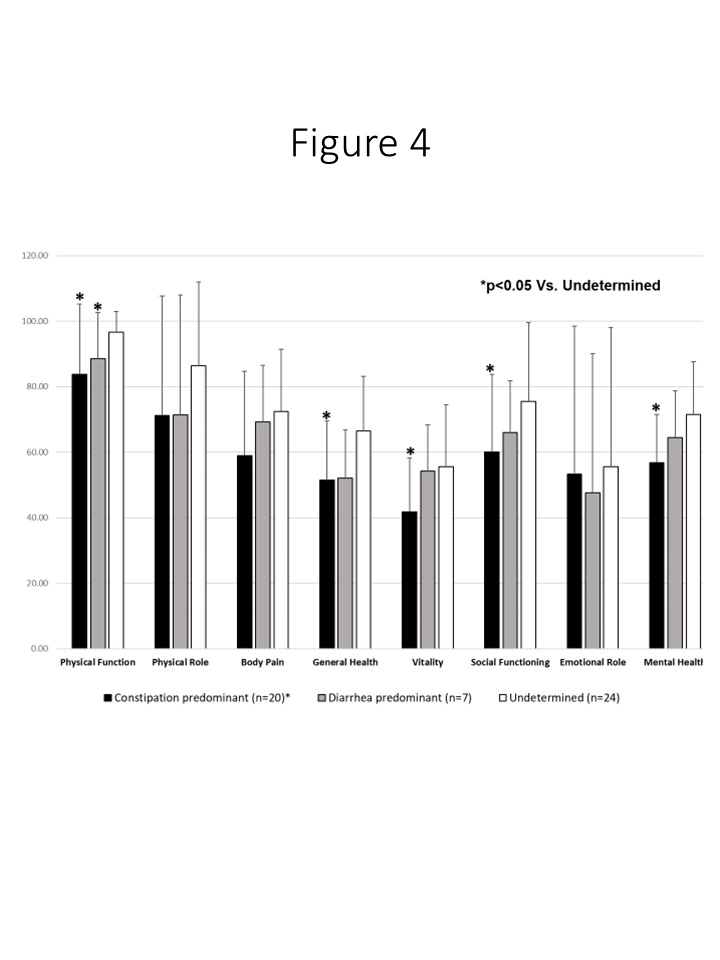
 **Supplementary figure C.** General Quality of life (SF-36) of women with irritable bowel syndrome (IBS) compared by subtypes.
